# Sacroillite tuberculeuse: à propos de deux cas

**DOI:** 10.11604/pamj.2016.25.69.10428

**Published:** 2016-10-04

**Authors:** Ismaël Diallo, Joëlle Tiendrébéogo Zabsonré, Bénilde Marie Ange Tiemtoré Kambou, Apoline Kongnimissom Sondo, Yempabou Sagna, Dieu-Donné Ouédraogo

**Affiliations:** 1 Service de Médecine Interne, Hôpital de jour du Centre Hospitalier Universitaire Yalgado Ouédraogo, Ouagadougou, Burkina Faso; 2 Service de Radiologie et d’Imagerie Médicale du Centre Hospitalier Universitaire Yalgado Ouédraogo, Ouagadougou, Burkina Faso; 3 Service de Maladies Infectieuses du Centre Hospitalier Universitaire Yalgado Ouédraogo, Ouagadougou, Burkina Faso

**Keywords:** Sacroillite tuberculeuse, VIH, Burkina Faso, Tuberculous sacroiliitis, HIV, Burkina Faso, Africa

## Abstract

La sacroiliite tuberculeuse est rare et de diagnostic difficile. Les auteurs rapportent deux cas. Il s'agissait dans le premier cas d'une patiente de 40 ans ayant une infection à VIH ; le diagnostic a été histologique après une biopsie chirurgicale. Le second cas a concerné un patient de 25 ans vivant en milieu carcéral chez qui le diagnostic a été établi sur la base des arguments cliniques, biologiques, radiologiques et l'efficacité du traitement ; l'intradermoréaction à la tuberculine était phlycténulaire. Le scanner a été indispensable au diagnostic lésionnel en montrant une érosion des berges et des abcès des parties molles. Le traitement a été médical et a fait appel aux antituberculeux.

## Abstract (en)

Tuberculous sacroiliitis is rare and of difficult diagnosis. We here report two cases. The first patient was a 40-year old woman with HIV infection; the diagnosis was based on histological evaluation after surgical biopsy. The second patient was a 25-year old man living in prison whose diagnosis was established on the basis of clinical, biological, radiological reasonings and of the effectiveness of the treatment; tuberculin intradermal reaction was phlyctenular. CT scan was essential to establish the lesion diagnosis by showing edge erosion and soft tissue abscess The patients received medical and anti-tuberculosis treatment.

## Introduction

La tuberculose connaît une recrudescence dans les pays en développement en raison de la pandémie du SIDA. Le rachis est la localisation ostéo-articulaire la plus souvent rapportée. L'atteinte des articulations sacro-iliaques est peu fréquente ne représentant qu'environ 10 % des tuberculoses ostéo-articulaires [1]. Son diagnostic reste difficile en raison de son expression clinique le plus souvent lombaire [1–3]. Nous rapportons deux cas de sacroiliite tuberculeuse et insistons sur les difficultés diagnostiques.

## Patient et observation

**Figure 1:**
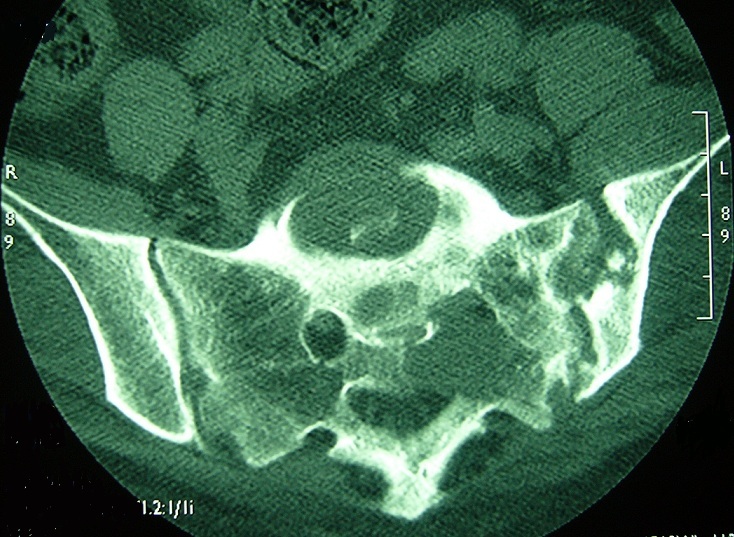
Coupe axiale sans injection (TDM du bassin): érosion des bords de l’articulation sacro-iliaque gauche prédominant au niveau du versant iliaque et ostéolyse sacrée s’étendant au trou sacré homolatéral

**Figure 2:**
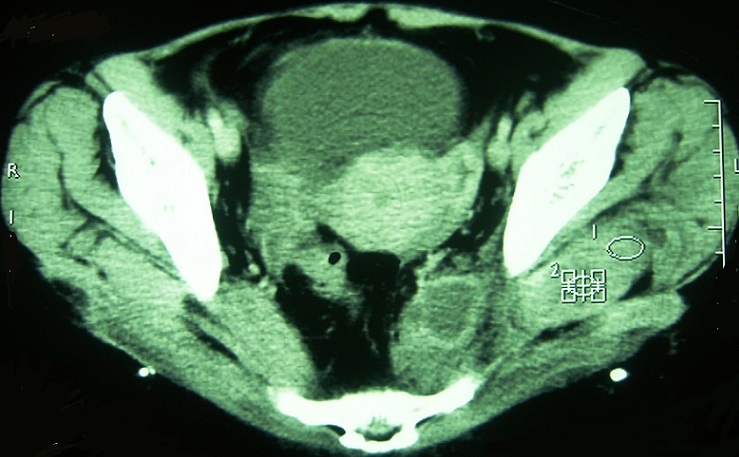
Coupe axiale avec injection de produit de contraste (TDM du bassin): infiltration du pisiforme gauche avec prise de contraste en cocarde témoignant de la présence d’un abcès du muscle

**Observation 1 :** une patiente de 40 ans sous traitement par Zidovudine, Lamivudine, et Efavirenz depuis 4 mois pour une infection à VIH de type 1, a été admise en hospitalisation pour une lombofessalgie gauche inflammatoire et invalidante d'installation insidieuse et progressive depuis 2 à 3 mois. Cette même symptomatologie avait récemment motivé une hospitalisation dans un service de neurologie où le diagnostic de lombosciatique gauche commune avait été posé. A l'interrogatoire, une vaccination par le BCG pendant l'enfance était rapportée. L'examen physique trouvait une fièvre chiffrée à 38°C chez une patiente ayant une impotence fonctionnelle relative du membre inférieur gauche ; la manœuvre d'écartement des sacro-iliaques était très douloureuse ; les tests de Fabere (flexion, abduction et rotation externe) et de Glaensen (extension de la hanche) étaient positifs. La palpation des apophyses épineuses des vertèbres lombaires était sans particularité. La recherche du signe de Lasègue était impossible en raison de la douleur. Le reste de l'examen était sans particularité. Les examens complémentaires montraient une leucocytose à 7600 pa rmm^3^ avec des polynucléaires neutrophiles à 5400/rmm^3^, des lymphocytes à 1400/mm^3^ et une anémie normocytaire, normochrome avec un taux d'hémoglobine à 8,3 g/dl ; les plaquettes étaient à un taux de 434 000/mm^3^. La vitesse de sédimentation était de 12 mm à la 1ère heure et la CRP à 12 mg/l. Le taux de CD4 était de 125 cellules/microlitre. Le bilan hépatique et la fonction rénale était sans particularité. La radiographie du bassin montrait une sacroiliite gauche confirmée par le scanner qui mettait en évidence d'importantes érosions de l'articulation sacro-iliaque gauche et du corps du sacrum et de multiples abcès localisés dans les muscles iliaques, fessiers et du pisiforme gauche (Figure 1 et Figure 2). La radiographie des poumons et du rachis lombaire était normale. La recherche de BAAR dans les crachats et dans le liquide de tubage gastrique était négative. L'étude histologique d'une pièce de biopsie de la lésion mettait en évidence un granulome gigantocellulaire avec un centre nécrotique posant le diagnostic d'une sacroiliite gauche tuberculeuse. L'étude bactériologique de la pièce n'a pas été faite. Une antibiothérapie antituberculeuse (isoniazide 5 mg/Kg/J, rifampicine 10 mg/Kg/J, pyrazinamide 30 mg/Kg/J, ethambutol 20 mg/Kg/J pendant deux mois puis rifampicine et isoniazide aux mêmes doses pendant 10 mois supplémentaires) a été réalisée. La fièvre et les douleurs ont régressé dès la première semaine. Le contrôle à un mois de traitement montrait une CRP à 6 mg/dl. Après 12 mois de traitement et un recul de 2 ans, il n'a pas été rapporté de récidive.

Observation 2 : Un homme de 25 ans, vivant en milieu carcéral, a été admis en hospitalisation pour une douleur inflammatoire et insidieuse de la hanche gauche évoluant depuis 2 semaines et d'une impotence fonctionnelle du membre inférieur gauche. Le patient rapportait un tabagisme chiffré à 5 paquets-années et un alcoolisme 10 mois avant son hospitalisation et une vaccination par le BCG à l'âge de 5 ans. L'examen physique montrait une fièvre à 37,8°C, une tuméfaction douloureuse et chaude au niveau de la fesse gauche. Le taux de leucocytes était à 10400/mm^3^avec une anémie à 8,7 g/dl microcytaire hypochrome. Le taux de plaquettes était à 229000/mm^3^. La vitesse de sédimentation était de 20 mm à la première heure et la CRP à 15 mg/dl. La sérologie rétrovirale (VIH) était négative. Le reste du bilan biologique était sans particularité. La radiographie du bassin était normale. Le scanner du bassin réalisé en raison de la persistance de la symptomatologie, a montré une sacroiliite gauche et de multiples abcès du psoas gauche. La radiographie du rachis lombaire et des poumons était normale. L'intradermoréaction à la tuberculine (tubertest 5UI) était phlyctenulaire. Le diagnostic d'une sacroiliite tuberculeuse gauche a alors été retenu et un traitement antituberculeux institué pendant 12 mois avec succès. Trois ans après, une fusion de l'articulation sacroiliaque a été observée.

## Discussion

La sacroiliite tuberculeuse (SIT) est de diagnostic difficile. Richter et al. ont rapporté que 92 % des patients ayant une SIT ont été initialement traités pour une lomboradiculalgie [4]. Cependant cette douleur est rarement associée à des signes neurologiques tels que les paresthésies, les dysesthésies ou des radiculalgies vraies [5, 6]. Ailleurs, la symptomatologie peut se limiter à une lombalgie [7]. L'intensité de la douleur et l'impotence fonctionnelle qui en résultent doivent constituer un signe d'appel. La notion d'un contage tuberculeux ou une histoire récente de tuberculose constituent une aide diagnostique précieuse [8], de même qu'une fébricule vespérale ou une hypersudation nocturne [1]. Les examens biologiques de routine montrent souvent un syndrome inflammatoire non spécifique (élévation de la vitesse de sédimentation et de la CRP) qui n'a aucun intérêt diagnostique mais permet de suivre l'évolution de la maladie sous traitement [1, 8]. Cette vitesse de sédimentation et la CRP ne semblent pas aussi élevées qu'au cours des sacroiliites à germes pyogènes [8]. La radiographie du bassin montre une irrégularité d'une articulation sacroiliaque dans les cas évolués [1]. Le scanner ou l'IRM du bassin confirment ou affirment le diagnostic lésionnel comme c'est le cas de notre seconde observation, en montrant des abcès des parties molles et une atteinte des structures osseuses voisines ; ces aspects radiographiques ne sont pas l'apanage des SIT et des lésions métastasiques [8], brucelliennes [9] doivent être discutées. L'IRM n'a été réalisée chez aucun de nos patients. Sa supériorité sur le scanner a cependant été rapportée par deux études [10, 11], en étant plus sensible dans le type de lésion et surtout en permettant un diagnostic précoce. L'intradermoréaction à la tuberculine est une aide au diagnostic chez les patients n'ayant pas une immunodépression comme c'est le cas de notre seconde observation [6]. Dans les pays à forte endémicité comme le nôtre, l'IDR devrait être franchement phlycténulaire en raison du contact fréquent avec le bacille de Koch. Le diagnostic de l'étiologie tuberculeuse est histologique (en présence d'un granulome gigantocellulaire avec une nécrose centrale) ou microbiologique (une culture positive sur milieu de Lowenstein-Jensen, de Coletsos ou en milieu liquide). La biopsie des lésions dans notre première observation a permis un diagnostic histologique tandis que dans le second cas, le diagnostic était présomptif devant un faisceau d'arguments. Neuf des 22 cas de SIT rapportés par Barajima en Tunisie ont été diagnostiqués sur la base d'arguments cliniques, biologiques, radiologiques et l'efficacité du traitement antibacillaire [2]. Notre première observation était associée à une infection à VIH. Le diagnostic de tuberculose impose en Afrique subsaharienne une recherche de l'infection à VIH en raison de leur association fréquente. Le traitement est basé sur une antibiothérapie antituberculeuse de longue durée (12 à 18 mois) [7, 12] cependant des protocoles plus courts (six à neuf mois) semblent également efficaces [2, 13]. Le pronostic de la SIT est favorable même en l'absence d'un traitement chirurgical comme c'est le cas de nos observations [14]. L'évolution finale se fait après traitement vers l'ankylose de l'articulation atteinte.

## Conclusion

Une SIT doit être évoquée devant des lombofessalgies hyperalgiques, confinant le patient au lit et interdisant toute mobilisation du membre inférieur en zone d'endémie tuberculeuse ou chez un patient immunodéprimé. Une radiographie du bassin normale doit inciter à la réalisation d'un scanner des articulations sacro-iliaques en l'absence d'IRM. Le traitement est conservateur et fait appel aux anti-bacillaires.
